# Extending the DIDEO ontology to include entities from the natural product drug interaction domain of discourse

**DOI:** 10.1186/s13326-018-0183-z

**Published:** 2018-05-09

**Authors:** John Judkins, Jessica Tay-Sontheimer, Richard D. Boyce, Mathias Brochhausen

**Affiliations:** 1 Department of Biomedical Informatics, University of Arkansas for Medical Sciences, Little Rock, AR USA; 2 School of Pharmacy, University of Washington, Seattle, WA USA; 3 Department of Biomedical Informatics, University of Pittsburgh, Pittsburgh, PA USA

**Keywords:** Biomedical ontologies, OWL, Pharmaceuticals, Pharmacokinetics, Drug-drug interactions, Natural product-drug interactions

## Abstract

**Background:**

Prompted by the frequency of concomitant use of prescription drugs with natural products, and the lack of knowledge regarding the impact of pharmacokinetic-based natural product-drug interactions (PK-NPDIs), the United States National Center for Complementary and Integrative Health has established a center of excellence for PK-NPDI. The Center is creating a public database to help researchers (primarly pharmacologists and medicinal chemists) to share and access data, results, and methods from PK-NPDI studies. In order to represent the semantics of the data and foster interoperability, we are extending the Drug-Drug Interaction and Evidence Ontology (DIDEO) to include definitions for terms used by the data repository. This is feasible due to a number of similarities between pharmacokinetic drug-drug interactions and PK-NPDIs.

**Methods:**

To achieve this, we set up an iterative domain analysis in the following steps. In Step 1 PK-NPDI domain experts produce a list of terms and definitions based on data from PK-NPDI studies, in Step 2 an ontology expert creates ontologically appropriate classes and definitions from the list along with class axioms, in Step 3 there is an iterative editing process during which the domain experts and the ontology experts review, assess, and amend class labels and definitions and in Step 4 the ontology expert implements the new classes in the DIDEO development branch. This workflow often results in different labels and definitions for the new classes in DIDEO than the domain experts initially provided; the latter are preserved in DIDEO as separate annotations.

**Results:**

Step 1 resulted in a list of 344 terms. During Step 2 we found that 9 of these terms already existed in DIDEO, and 6 existed in other OBO Foundry ontologies. These 6 were imported into DIDEO; additional terms from multiple OBO Foundry ontologies were also imported, either to serve as superclasses for new terms in the initial list or to build axioms for these terms. At the time of writing, 7 terms have definitions ready for review (Step 2), 64 are ready for implementation (Step 3) and 112 have been pushed to DIDEO (Step 4). Step 2 also suggested that 26 terms of the original list were redundant and did not need implementation; the domain experts agreed to remove them. Step 4 resulted in many terms being added to DIDEO that help to provide an additional layer of granularity in describing experimental conditions and results, e.g. transfected cultured cells used in metabolism studies and chemical reactions used in measuring enzyme activity. These terms also were integrated into the NaPDI repository.

**Conclusion:**

We found DIDEO to provide a sound foundation for semantic representation of PK-NPDI terms, and we have shown the novelty of the project in that DIDEO is the only ontology in which NPDI terms are formally defined.

## Background

Concomitant use of prescription drugs and natural products, including vitamin, mineral, or herbal supplements, is a frequent occurrence. The high prevalence of natural products (NP) usage raises concerns about the potential impact on drug effectiveness and toxicity from natural product drug interactions. Pharmacokinetic-based natural product-drug interactions (PK-NPDIs) are of particular concern because their potential impact on drug effectiveness or toxicity is often unknown.

To provide evidence-based information regarding purported PK-NPDIs, a new Center of Excellence for PK-NPDI Research was established by the United States National Center for Complementary and Integrative Health (grant number U54 AT008909). The Center is creating a publicly accessible database where researchers can access scientific results, raw data and recommended approaches to optimally assess the clinical significance of PK-NPDIs. One of the requirements of the repository is that it represent data in a semantically rich and interoperable way.

There have been previous efforts to provide ontological representation of this domain. We reviewed existing ontologies to consider re-use. Searching the NCBO Bioportal [1] retrieves the Natural Product Ontology (NATPRO) [2], which we consider a potentially relevant resource for our project. According to an article by the person whose name appears as contact on the Bioportal site for NATPRO [3], it seems that the use case under which NATPRO was developed was mining information about natural products to bring new ideas to drug development, which is similar to our own goals. NATPRO was submitted to the NCBO Bioportal in 2012 and, at the time of writing, there are no reported updates. The NCBO BioPortal landing page for NATPRO does not provide additional documentation or links to resources (e.g. a code repository). The ontology contains 9465 classes and 22012 individuals, is based on the BioTop ontology [4], a top-domain ontology for the life sciences, re-uses classes from Chemical Entities of Biomedical Interest (ChEBI) [5] and cross-references medical conditions with the Human Disease Ontology [6].

The biggest hurdle against re-using NATPRO is the lack of textual definitions for the classes. One of the key use cases for standardizing terms in the PK-NPDI data repository is to help users easily locate studies of interest using queries that will include mention of study features. Textual definitions are key to supporting this use case. Of the 9465 classes that NATPRO provides, only five come with a textual definition, and even among those five, the ontology is inconsistent in the choice of annotation label. In one single case the following annotation is used: [http://purl.org/imbi/ru-meta.owl#definition]. Others have rdfs:comment for definitions. Many terms are annotated with the data property hasDefinition, but these annotations point to URIs as values that neither resolve nor communicate useful information. The lack of definitions or descriptions of classes leads to ambiguities that potential users find themselves unable to resolve. For example, under *ToxicEffect* we find *ArrowPoison*. The label suggests that this is a representation of the class of arrow poison. However, its superclass suggests that it is intended to represent the toxic effects created through arrow poison. The ambiguity gets amplified through the fact that the labels of other subclasses of *ToxicEffect* clearly identify that they represent an effect, e.g. *DefibrinogenatingEffect*. Providing textual definitions helps potential users to overcome these kinds of ambiguity.

The lack of textual definitions also hinders the interpretation and the validation of axioms provided by NATPRO. For example, the superclass to *ToxicEffect*, *BiologicalActivity*, does not have a textual definition but an axiom that expresses that only members of the class *ActivityMeasure* are valid measurements of BiologicalActivity. Since *ActivityMeasure* does also not have a textual definition, we have no way of validating whether that axiom is correct or not. Running a reasoner over the ontology and data annotated with it would assign everything that measures an instance of *BiologicalActivity* to be an instance of *ActivityMeasure*, no matter whether that would be sound or not. Object properties in NATPRO also lack definitions, even though they do appear in axiomatic statements denoting relationships between classes, e.g. “Cyanobacteria associatedWith only PathologicalCondition”. Such axioms can neither be evaluated as true nor even be interpreted.

Another hurdle against re-using NATPRO is that its focus is only on effects of NPs on the body rather than interactions between NPs and drugs. This is clear from the description of NATPRO in BioPortal as “An ontology for describing biological activities of natural products” and a publication whose author is listed as the contact in BioPortal for NATPRO [3]. A biological activity is defined in the publication as “a change of state of a target brought about by its interaction with a natural product”; a gene protein, cell and microorganism are provided as examples of a target. Therefore, there is no representation of entities that have to do with the experiments through which those biological activities are discovered and confirmed. This representation is crucial to our goal of providing rich semantics to data useful to assess significance of NPDIs. Because observations and assertions of mechanisms from a variety of experiments and clinical studies contribute to evidence of interactions [7], representation of all related entities allows this evidence to be traced and assessed using a reasoning system. Another shortcoming of NATPRO is its complete lack of activity in the past five years. The usefulness of an ontology developed for this domain depends on the extent of effort to keep the ontology updated as new data becomes available on the use and effects of natural products. Its usefulness also depends on the extent of documentation. This is especially problematic since it is not apparent how classes in the ontology having been given names such as “_DUMMY-FRAMES-METACLASS” are meant to be interpreted and used. The documentation that can be found is also out of date; the Bioportal site links to a site that no longer exists but was meant to provide references to elucidate the meaning of object properties in NATPRO. Thus, while NATPRO might provide quite an extensive term list relevant to our domain of interest, it lacks accessible documentation and does not provide the definitions or rich semantics required by our project. This not only makes NATPRO a poor starting point for our development, the representational problems described here also discourage use of individual classes from this ontology in future development of a logically consistent and semantically-rich representation of the natural product-drug interaction domain.

Spyrou and Lange reported on the development of a food-drug interaction ontology [8]. uc_FIDO is intended to serve as an unambiguous representation of food-drug interactions to be used by healthcare consumer-oriented knowledge sources. Of the 818 classes in uc_FIDO [9] none has a definition. Some of the design decisions described in the paper presenting uc_FIDO do not inspire confidence in the resulting ontology. The authors suggest that ingredients of foods are related to those foods using a subclass relation, e.g. in the case of *sourdough* and *all-purpose_flour* [8]. Given the set theoretical nature of OWL classes, this does not seem an appropriate representation. According to OWL, if A and B are subclasses, each member of A is also a member of B. It is not true that all members of the class *all-purpose_flour* are members of *sourdough*. In conclusion, we do not think that uc_FIDO provides sufficiently rigorous and rich semantics for our project. This not only makes uc_FIDO a poor starting point for our development, the representational problems described here also discourage use of individual classes from this ontology in future development of a logically consistent and semantically-rich representation of the natural product-drug interaction domain.

Since there are a number of similarities between pharmacokinetic drug-drug interactions and PK-NPDIs, we turned to the Drug-drug Interaction and Drug-Drug Interaction Evidence Ontology (DIDEO) [10] that provides terms and definitions relevant to the knowledge and evidence of potential drug-drug interactions [7]. DIDEO is coded in the Web Ontology Language (OWL2) and conforms to OBO Foundry principles [11], which were created to promote an environment of open availability, interoperability and reusability among ontologies for biosciences. DIDEO is undergoing active development. Each new class added to it is given a unique and specific definition; comments and examples are also often given in order to provide further information. Based on existing overlap and the rich semantics of DIDEO, we decided to extend DIDEO to include definitions for all terms used by the new PK-NPDI repository. In this paper, we report the state of the development and our methods to represent the domain of NPDI, including the review of ontologies potentially relevant to the field.

## Methods

The aim of our extension to DIDEO is coverage of the entire scope of pre-clinical and clinical experimental data for NPDIs. PK-NPDI data originates from study reports and published papers describing pre-clinical and clinical studies, in vivo or in vitro, involving NPs. Critical data from the experiments includes study metadata, descriptions of the included NPs and other chemical compounds used, experimental conditions, and resulting measurements. The pharmaceutical researchers serving as domain experts for this project assembled NaPDI terms with the intention of capturing descriptions of in vitro and clinical evaluations of natural product metabolism and transport and their potential for drug interactions with respect to inhibition and induction. Concepts for NaPDI terms were developed from drug interaction representations used in the University of Washington’s Metabolism and Transport Drug Interaction Database (DIDB) platform [12], which has served as a reliable source of drug interaction data for pharmaceutical researchers for over 15 years and has a large collection of manually curated experiments. As of today the DIDB has data from over 14,000 published citations and over 200 New Drug Applications (NDAs) that users can access, with new citations being added daily. Concepts were applied to and enriched for drug interaction studies involving natural products. Concepts were used to capture information such as study classification, test system or study population, experimental conditions or study design, study results, cut-off values, measured preclinical or clinical parameters, and variability. When possible, standard classification systems were used, such as NIH categorization of study population ethnicity and adverse event terms from Common Terminology Criteria for Adverse Events (CTCAE). Terms were products of extensive experience of the NaPDI project team in drug interaction knowledge representation and current drug interaction study practices.

PK-NPDI study data covers a finer granularity of experimental conditions and results than the existing DIDEO. Adding OWL representation of these parameters is guided by the annotation properties currently used in DIDEO. Table 1 shows the following annotation properties we frequently use to create a class representation in DIDEO. Label and Definition are required. User-centered definitions are entered using the Comment property. We also strive to provide equivalent class axioms and subclass axioms for the terms we add.

**Table 1:** Annotation properties required to create a class representation in DIDEO

Property	URI
Label	http://www.w3.org/2000/01/rdf-schema#label
Definition	http://purl.obolibrary.org/obo/IAO_0000115
Comment	http://www.w3.org/2000/01/rdf-schema#comment
Example of usage	http://purl.obolibrary.org/obo/IAO_0000112
Term editor	http://purl.obolibrary.org/obo/IAO_0000117
Alternative term	http://purl.obolibrary.org/obo/IAO_0000118

In representing the necessary entities for DIDEO, we aim to follow OBO Foundry principles. For our methodology, the most relevant are the requirement for textual definitions and following the naming conventions [11]. We also aimed to provide textual definition in a way that has become widely adopted among OBO Foundry ontologies and that has been outlined by Seppälä et al. [13]: the genus differentia form, which gives first the genus proximus (the immediate superclass) and then specifies properties of the defined class that set it apart from all classes with the same genus proximus. However, in our work with domain experts in other projects, we have found that this kind of definition is often less useful to domain experts. Hence, we use rdfs:comment to represent a domain expert-focused definition that has the same extension as the textual definition [14].

One principle of the OBO Foundry is that ontologies should be created with the capability to be used across multiple scientific communities [15]. Therefore, in order to avoid ambiguity, the name contributed by the domain experts may need to be expanded; e.g. a community might refer to a microtiter plate as a “plate” in its internal communication, but this name should be expanded to “microtiter plate” for an ontology label. We also follow the guidelines of the Ontology for Biomedical Investigation (OBI) in using singular rather than plural names for labels [16, 17]. These guidelines may lead us to choose a different label for a class than the one chosen by domain experts. In such a case, we preserve the name provided by domain experts as an alternative term in the ontology, so that either name may be entered in a search for the class.

The OBO Foundry also encourages the re-use of terms from other ontologies to facilitate collaboration. The need for consistency in importing these terms led to the creation of guidelines known as the Minimum Information to Reference an External Ontology Term (MIREOT) [18]. According to MIREOT guidelines, the minimum information required to integrate a term from an external ontology consists of the following URIs: the external ontology, the term itself and direct superclass.

To provide an ontology that is inline with the ontology development principles outlined above and the needs of our users in the NPDI domain, we set up an iterative domain analysis process that brings together both domain experts and ontology experts. In Step 1 the three NPDI domain experts provide a list of terms and descriptions for terms relevant to the Center of Excellence for PK-NPDI Research; in Step 2 an ontology expert checks whether an appropriate ontological representation already exists in an OBO Foundry ontology. If no such representation exists, the ontology expert selects an appropriate label for the term, provides a genus-differentia definition, and if possible a necessary and sufficient condition. In Step 3 the domain experts review the material provided by the ontology expert. If they find the material correct, they mark the term as ready for implementation, if not they push it back to Step 2 with suggestions for improvement. Finally, in Step 4 the ontology expert implements the term in the development version of DIDEO and pushing the term to its code repository [19]. The DIDEO curators turn development versions into releases on a regular basis.

In Step 1 the domain experts choose terms, write definitions for them and review each others’ work. Step 2 is accompanied by teleconferences between the domain experts and the ontology expert to clarify the terms and resolve questions that require domain knowledge. We found that after Step 2 and during Step 3 the domain experts also frequently edited the initial description of the term to optimize its content. We have decided to use those descriptions as user-centered definition of the classes in DIDEO and add them using the rdfs:comment annotation. Examples of usage were added to the OWL file where they were provided by the domain experts.

**Fig. 1:**
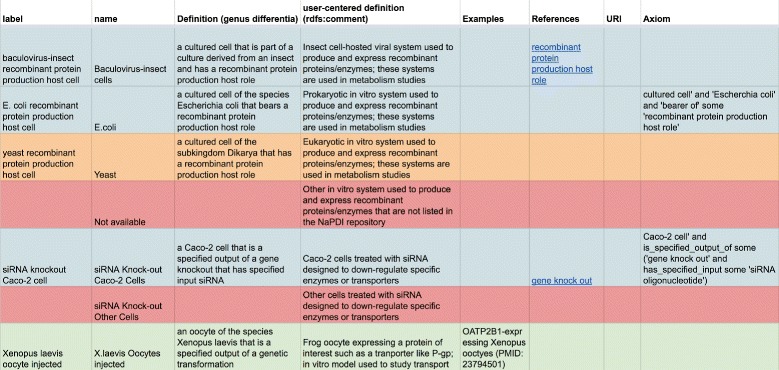
Screenshot of the DIDEO-NPDI working document, accessible for all domain experts and ontology experts in the project to view and edit online

We implemented this workflow using a Google Sheet table structure that is accessible to all domain experts and ontology experts involved in the process; a screenshot is shown in Fig. 1. The online document provides a system of different tables providing columns for the term and term descriptions from the NPDI experts. The tables also contain category names and questionnaire information as it appears in the domain experts’ data management system. For the ontology development process columns for “label”, “genus differentia definition”, “user-centered definition”, “examples of usage” are provided. One column is designated to report URIs of classes re-used from other ontologies. The DIDEO development strategy rests on the idea that well-built ontological representations from other ontologies should be re-used wherever possible. The ontologies re-used for the NPDI subproject are ChEBI [5], the Gene Ontology [15], OBI [16], and the Ontology for Medically Related Social Entities [20].

In addition to the system of tables, we introduced a color code to represent workflow progress on a specific term. Because each row of the spreadsheet is given to a distinct term and its annotations, each row is filled with a red, orange, yellow, green, or blue color to indicate progress on the definition of its term. Red is used to annotate terms which are still under consideration of the ontology expert and are not yet ready to be reviewed by the domain experts (Step 1). Yellow indicates that a term is ready for review through the domain experts (Step 2). Green indicates that a term is ready to be implemented in the OWL file (Step 3). Switching a term annotation from yellow to green can only be done by a domain expert (Step 1), ensuring they vet and approve a term before implementation in DIDEO. If the domain expert sees the need to revise the term, its definition or any other annotation, they will set the color to orange, which will trigger the ontology expert to revisit the term. Terms that have already been implemented in DIDEO are marked blue (Step 4). Once the term is in DIDEO, changes can still be requested using the DIDEO issue tracker [21]. This procedure of introducing each definition for vetting through an iterative process of review and approval ensures consensus is reached between the domain experts and the ontology expert that the definition conforms to both the meaning of the term as understood by the domain experts and the principles of the OBO Foundry and the OBI ontology from which many terms in DIDEO are imported.

Additional steps are taken toward providing compatibility between OWL representations of PK-NPDI data and vocabularies used with other pharmacological data sets. Terms, definitions and alternative terms in DIDEO are downloaded as a CSV file using OWL2TL [22] and converted to entries in the database terminology browser [23]. The browser shows the DIDEO terms (included imported terms) along with terms from terminologies organized by the standard vocabulary used by Observational Health Data Sciences and Informatics collaborative (OHDSI) [24], an organization that promotes the standardization of biomedical data across diverse sources. OHDSI developed the ATLAS web-based platform for exploring standardized vocabularies. The PK-NPDI center has developed a customization of ATLAS to complement the PK-NPDI evidence database. This allows easy access to natural-language definitions of terms for users as they browse PK-NPDI evidence entries, in addition to providing compatibility with other data sets.

## Results

Table 2 shows the development status of all terms from the NaPDI repository according to the development steps specified in the Methods section. This status is the result of approximately 250 hours of work by the three domain experts on this project. The initial domain analysis through the domain experts (Step 1) resulted in a total of 305 terms and term descriptions provided by the domain experts. Further domain analysis increased this number to 344. During Step 2 we found that 9 terms out of the 344 already existed in DIDEO and 6 terms existed in other OBO Foundry ontologies. We imported these 6 terms individually into DIDEO using a MIREOT plugin developed at the University of Arkansas for Medical Sciences [25]. In addition, we imported terms from multiple OBO Foundry ontologies as they became necessary as superclasses for new classes or for providing axioms for new classes using the same plugin.

**Table 2:** Number of terms in each development step

Development status	Development step completed	Number of terms
To do	Step 1	119
Already existing in DIDEO	Step 2	9
Definition provided	Step 2	7
Ready for OWL implementation	Step 3	64
In DIDEO	Step 4	112
Imported from other ontologies	Step 4	7
**Total terms to develop**		**318**
Redundant terms		26
**Total terms provided by domain experts**		**344**

Bold text gives totals

For example, ChEBI and the Molecular Process Ontology contains terms for chemical species and chemical reactions, respectively, that we used to build definitions and axioms for new chemical reaction terms that resulted from the domain analysis. Table 3 shows the source ontologies from which individual terms were re-used and gives the number of classes or object properties imported using MIREOT for each source ontology. Step 2 also suggested that 26 of the terms initially provided were redundant. The ontology editor verified that with the domain experts and, in agreement with them, removed them from the implementation plan.

**Table 3:** Number of individual terms imported into DIDEO using MIREOT

Ontology	Classes and object properties
Apollo Structured Vocabulary	1
Cell Line Ontology	5
Cell Ontology	2
Chemical Entities of Biological Interest	37
Chemical Methods Ontology	2
eagle-i resource ontology	8
Gene Ontology	5
Molecular Process Ontology	7
NCBI organismal classification	3
Ontology for Biomedical Investigations	27
Relations Ontology	3
The Statistical Methods Ontology	2

Table 4 gives examples of terms that have been created newly for DIDEO as part of the NPDI integration process and that are the outcome of Step 4. The examples are terms that are crucial for reaching our goal of providing additional layers of granularity necessary to represent experimental conditions and results. This is meeting the requirement from the NPDI integration as specified in the Methods section. The omeprazole 5-hydroxylation reaction is useful in measuring the activity of the CYP2C19 enzyme [26] and is an example of representations of chemical reactions relevant to both DDI and NPDI. Other terms newly added to DIDEO provide representations of systems of cultured cells or microsomal fractions. These are frequently used in in vitro experiments as models to study biological metabolism and transport.

**Table 4:** Example new classes in DIDEO for NPDI

Label	Textual definition	ID (DIDEO_)	Axiom
E. coli recombinant protein production host cell	a cultured cell of the species Escherichia coli that bears a recombinant protein production host role	00000169	‘cultured cell’ and ‘Escherchia coli’ and ‘bearer of’ some ‘recombinant protein production host role’
siRNA knockout Caco-2 cell	a Caco-2 cell that is a specified output of a gene knockout that has specified input siRNA	00000188	‘Caco-2 cell’ and is_specified_output_of some (‘gene knock out’ and has_specified_input some ‘siRNA oligonucleotide’)
293 transfected cell	a 293-derived cell that is a specified output of a transfection	00000189	‘293-derived cell’ and is_specified_output_of some transfection
omeprazole 5-hydroxylation	a hydroxylation that has omeprazole as input and has 5-hydroxyomeprazole as output	00000175	hydroxylation and ‘has input’ some omeprazole and ‘has output’ some 5’-hydroxyomeprazole
7-ethoxyresorufin O-deethylation	a deethylation that has 7-ethoxyresorufin as input and resorufin as output	00000172	deethylation and ‘has input’ some 7-ethoxyresorufin and ‘has output’ some resorufin

**Fig. 2:**
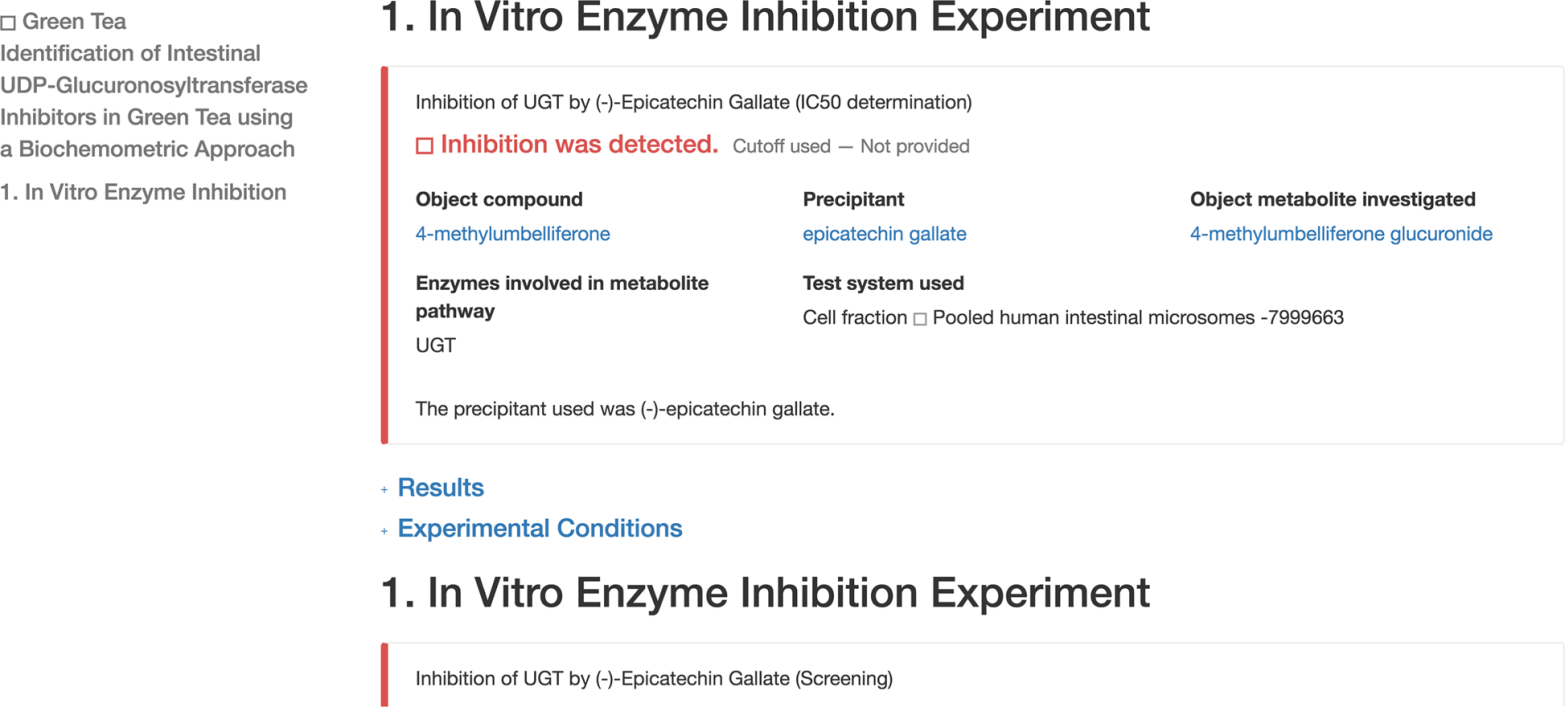
Screenshot of the NaPDI repository showing a green tea study that provides links to several term definitions

All DIDEO terms were integrated into the OHDSI-based standard vocabulary system used by the NaPDI repository. Progress has been made toward enabling users to view term definitions as they review studies. For example, Fig. 2 shows a screenshot of a green tea study [27] that provides links that users will be able to click to view term definitions and relationships; e.g. the link in the ID number next to the phrase “pooled human intestinal microsomes” will link to information on the DIDEO class ‘pooled human intestinal microsomal fraction’. Work is ongoing to improve the presentation of links within the repository and usability of the terminology explorer for NaPDI users.

The initial domain analysis providing the 305 terms and the continuation of the analysis increasing the number of terms to 344 serves to indicate progress toward our aim of covering the whole scope of NPDI pre-clinical and clinical experimental data.

## Discussion

One important challenge was ensuring definitions have the correct level of specificity. Step 2 revealed some user-centered definitions that needed more information, either to allow the ontology expert to properly categorize the term, viz. to pick the *genus proximum*, or to allow writing about the distinguishing properties (*differentia*) of the entities in question. The most important tools to address these challenges and communicate solutions were the email discussions and teleconferences scheduled every other week between the ontology expert and domain experts.

These issues were addressed iteratively, using the communication tools specified above. Changes to the initial description were added to the Google Sheet table. User-centered definitions also frequently provided a generous amount of information beyond the genus and the distinguishing properties, including how the cell system is used in experiments. Examples of these can be seen in Fig. 1. While we retained all information in the domain expert provided description, we did not use the material going beyond essential characteristics for editing the genus differentia definition.

Another issue that we encountered was how to label the entities in the ontology and how that label was related to the name provided by the domain experts, this name in some cases being very general in meaning, e.g. in the case of “dilution factor”. Checking against the user-centered definition, it becomes obvious that what is meant here is the dilution factor of the pre-incubation mixture in an in vitro experiment studying inhibition of an enzyme. Using highly general labels to refer to something that is highly context specific is commonly done and often creates no ambiguity. However, due to the open nature of the OBO Foundry, terms need to be labeled in such a way that importing and integrating terms from multiple domains (viz. multiple contexts) is facilitated. This leads to the principle that a label should adequately address the same level of specificity as the definition in order to avoid confusion. Similarly, a decision was needed when given a plural name. Resolving this was especially challenging for names containing the word “microsomes”. The meaning of “microsomes” is clear to the domain experts; however, defining a single microsome proved problematic since it is not referred to in the singular and could be one of many types of objects resulting from cell lysis. Our solution was to provide the phrase “microsomal fraction” for labels, defining the phrase as the pellet resulting from centrifugation of an S9 fraction.

Another challenge was representing the hierarchy of measurements, ratios of measurements and ratios of ratios in the list of in vitro measurements provided by the domain experts. Imported terms from OBI allow us to represent the output data of measurements such as length and temperature. However, calculations deriving from these measurements require more complex treatment, especially time rates. As an example, ‘transporter substrate accumulation rate’, defined by the domain experts as “the rate in which a substrate is either taken into a given number of cells or passes through an amount of transporter protein”, is described in a subclass axiom as a ‘rate measurement datum’ that ‘has part’ some ‘time measurement datum’. This manner of representation provides useful input to a reasoning system without forcing the decision of how to represent the rate itself apart from the measurement or calculation of the rate. Representation of measurements has been well-established, but the question of how to represent the rate itself is quite controversial and answered differently among OBO Foundry ontologies. For example, the OBO community is divided over whether the velocity of an object is a quality that inheres in the object or a part of the process of the motion of the object.

We also made a minor change to the way DIDEO is developed and updated in its online repository. At the beginning of this project, NaPDI terms were added to the development branch of DIDEO on GitHub, which is periodically pushed to a release version. However, it became necessary to create a development branch “john” separate from the main development branch, in order to prevent conflicts between activity on the NaPDI extension and other activity on DIDEO. It will be merged into the main development branch periodically.

Future work will also focus on leveraging DIDEO terms to enable the export of NaPDI repository studies in a standardized format, and integration with data from drug-drug interactions annotated in scientific publications and drug product labels [28]. In addition to that we will develop a test to determine whether our extension to DIDEO has met our coverage aims. Another aspect that we will address in future development cycles is how to represent lifestyle factors such as alcohol consumption and marijuana use. These are categorized by the domain experts into regular, occasional or nonexistent (for marijuana use) and healthy, unhealthy or nonexistent (for alcohol consumption). However, looking at the general use of such categorizations, there is a disagreement on boundaries separating these categories and what defines a non-smoker or non-drinker. Multiple data are necessary to thoroughly characterize alcohol consumption: frequency of drinking events, number of drinks during an event (or blood alcohol level) and how long someone needs to abstain to become a non-drinker. Any terms should be defined with regard to these components. Similar distinctions can be made to separate light, heavy and non-smokers (tobacco or marijuana). Viewed against the OBO Foundry principles stressing the importance of reusability and appropriate scope, contextual definitions of such categories would be problematic.

The utility of the ontology is driven by the use cases for the NaPDI repository mentioned in the Introduction section: the first being interoperability between the data it contains and data in other drug repositories and the second being data search and retrieval capability. Once definitions are complete, studies can be conducted to evaluate this utility. This extension to DIDEO is by no means limited to the aforementioned use cases; we are capable of extending DIDEO further to accomodate additional cases.

## Conclusion

Though in an early stage, our extension to DIDEO is progressing toward our goal of including all terms used in the Center’s PK-NPDI database and providing definitions for them. We have also shown that DIDEO provides a sound foundation on which to achieve our goal, as it follows OBO Foundry principles that include the re-use of terms from other medical ontologies such as OBI. We have also demonstrated the novelty of this project in that, while both an NP ontology and a food-drug interaction ontology exist, they do not meet the project’s requirements for complete textual definitions and rich semantics. Moreover, ours is the only project to build an ontology for NPDIs with formally-defined terms. We maintain this extension to DIDEO as an ongoing, open and community-driven effort. Plans are also underway to implement a website to facilitate further review of the user-centered descriptions by a larger group of domain experts as we continue adding to the ontology. This will further ensure no remaining ambiguity in definitions for researchers using the ontology and repository. Discussions and comments are welcome at the project’s GitHub site [19] or on the Drug Interaction Knowledge Base public forum [29].
